# Supplementary material for: ABCC1 and glutathione metabolism limit the efficacy of BCL-2 inhibitors in acute myeloid leukemia
Source: Nat Commun. 2023 Sep 19;14:5709. doi: 10.1038/s41467-023-41229-2 (PMC10509209; doi:10.1038/s41467-023-41229-2)
Supplement: Supplementary file 1 — Supplementary Information [file 41467_2023_41229_MOESM1_ESM.pdf]

SUPPLEMENTARY Information

ABCC1 and glutathione metabolism limit the efficacy of BCL-2 inhibitors in acute myeloid leukemia

Jessica Ebner*[1], Johannes Schmoellerl*[1,2], Martin Piontek[1], Gabriele Manhart[1], Selina Troester[1], Bing Z. Carter[3], Heidi Neubauer[4], Richard Moriggl[4], Gergely Szakács[5,6], Johannes Zuber[2,7], Thomas Köcher[8], Michael Andreeff[3], Wolfgang R. Sperr[9,10], Peter Valent[9,10], Florian Grebien[1,11]

[1] Institute for Medical Biochemistry, University of Veterinary Medicine Vienna, Austria

[2] Research Institute of Molecular Pathology (IMP), Vienna BioCenter (VBC), Vienna, Austria

[3] Section of Molecular Hematology and Therapy, Department of Leukemia, The University of Texas MD Anderson Cancer Center, TX, Houston, USA

[4] Institute for Animal Breeding and Genetics, University of Veterinary Medicine Vienna, Austria

[5] Center for Cancer Research, Medical University Vienna, Austria

[6] Institute of Enzymology, Research Centre of Natural Sciences, Eötvös Loránd Research Network, Budapest, Hungary

[7] Medical University of Vienna, Vienna, Austria

[8] Vienna BioCenter Core Facilities, Vienna BioCenter, Vienna, Austria

[9] Department of Internal Medicine I, Division of Hematology and Hemostaseology, Medical University of Vienna, Austria

[10] Ludwig Boltzmann Institute for Hematology and Oncology, Medical University of Vienna, Austria

[11] St. Anna Children's Cancer Research Institute (CCRI), Vienna, Austria

* These authors contributed equally: Jessica Ebner and Johannes Schmoellerl

Correspondence:

Florian Grebien
Institute for Medical Biochemistry, University of Veterinary Medicine Vienna, 1210 Vienna, Austria
florian.grebien@vetmeduni.ac.at

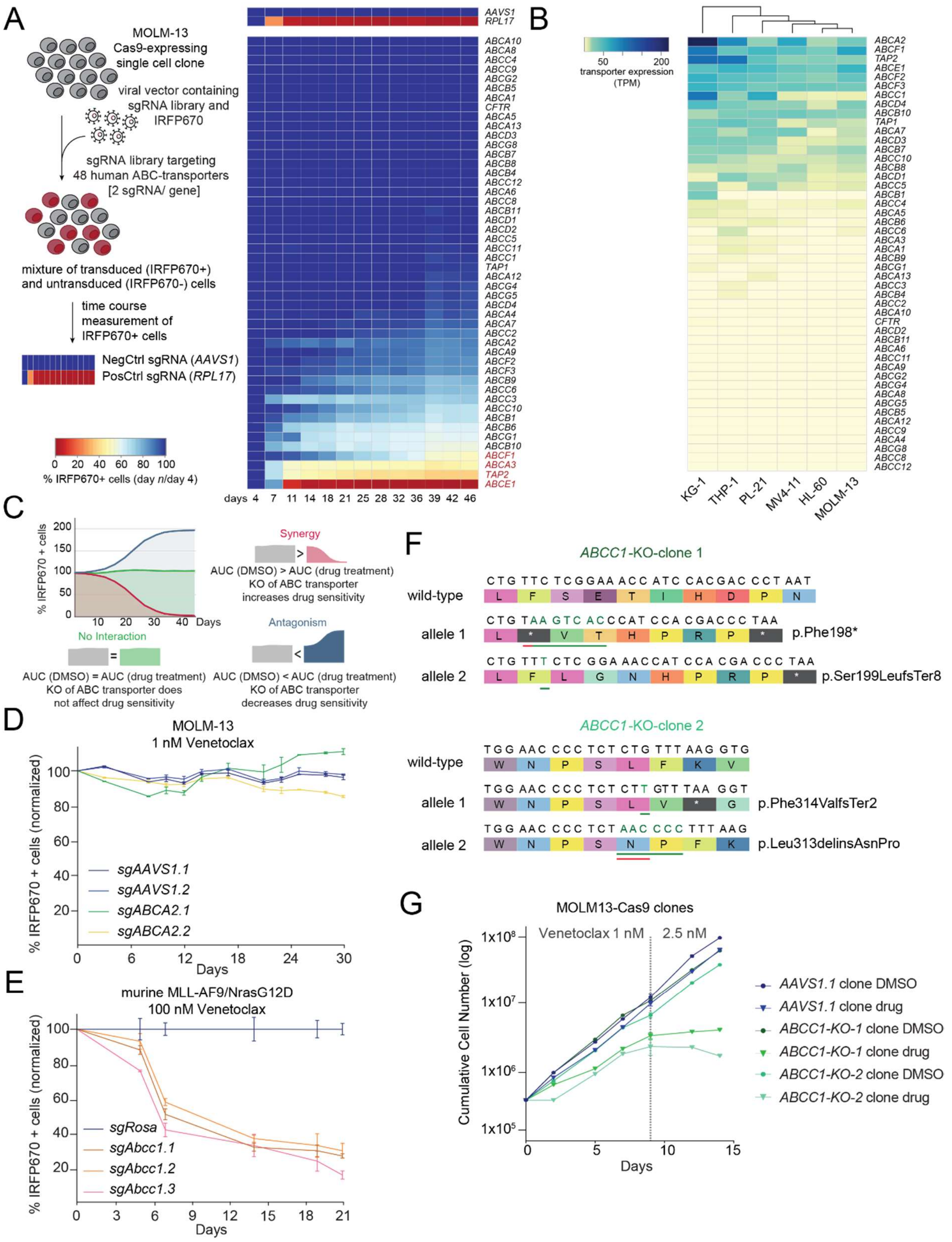

Supplementary Figure 1: *ABCC1* expression modulates Venetoclax sensitivity in AML cells

(A) Schematic representation of the CRISPR/Cas9-based competitive proliferation assay. Each *ABC transporter* gene is targeted with two sgRNAs in Cas9-expressing MOLM-13 cells. Cells were divided and the percentage of sgRNA-expressing cells (IRFP670^{+}) were monitored

over time (left). The corresponding gating strategy for IRFP670$^+$ cells is depicted in Supplementary Figure (6A). Cells transduced with sgRNAs targeting *AAVS1* served as negative control (safe harbor locus), whereas cells transduced with sgRNAs targeting *RPL17* served as positive control for depletion (essential gene). The heatmap is depicting the mean percentage of sgRNA expressing cells normalized to day 4 after transduction (right). n=4 experimental replicates with 2 different sgRNAs per gene. (B) Heatmap depicting the expression (transcripts per million, TPM, from the Ordino database) of all 48 human *ABC transporters* in the 6 indicated human AML cell lines (KG-1, THP-1, PL-21, MV4-11, HL-60, MOLM-13). (C) The percentage of sgRNA-expressing cells (IRFP670$^+$) were monitored over time and the area under the curve (AUC) was determined for each sgRNA as indicated and compared between DMSO and drug treated samples. (D) Competitive proliferation assay of MOLM-13-Cas9 cells treated with 1 nM Venetoclax for 30 days. Percentages of sgRNA/IRFP670+ cells were normalized to day 0 of treatment and to DMSO controls. Data are presented as mean values ± SD. n=2 experimental replicates. (E) Competitive proliferation assay of murine MLL-AF9/NRasG12D (RN2) –Cas9 cells treated with 100 nM Venetoclax for 21 days. Percentages of sgRNA/IRFP670+ cells were normalized to day 0 of treatment, to sg*Rosa* (negative control) and to DMSO controls. Data are presented as mean values ± SD. n=3 experimental replicates. (F) Sequence changes on both alleles of sg*ABCC1* targeted Cas9-expressing MOLM-13 clones (*ABCC1-KO-1/2*) compared to the wild-type sequence determined by TA cloning and Sanger sequencing. (G) Growth curves of MOLM-13-Cas9 knockout clones (*AAVS1.1, ABCC1-KO-1, ABCC1-KO-2)* treated with DMSO or Venetoclax (increasing concentrations 1 and 2.5 nM). Data are presented as mean values ± SD. n=3 experimental replicates. (A,B,D-G) Source data are provided as a Source Data file.

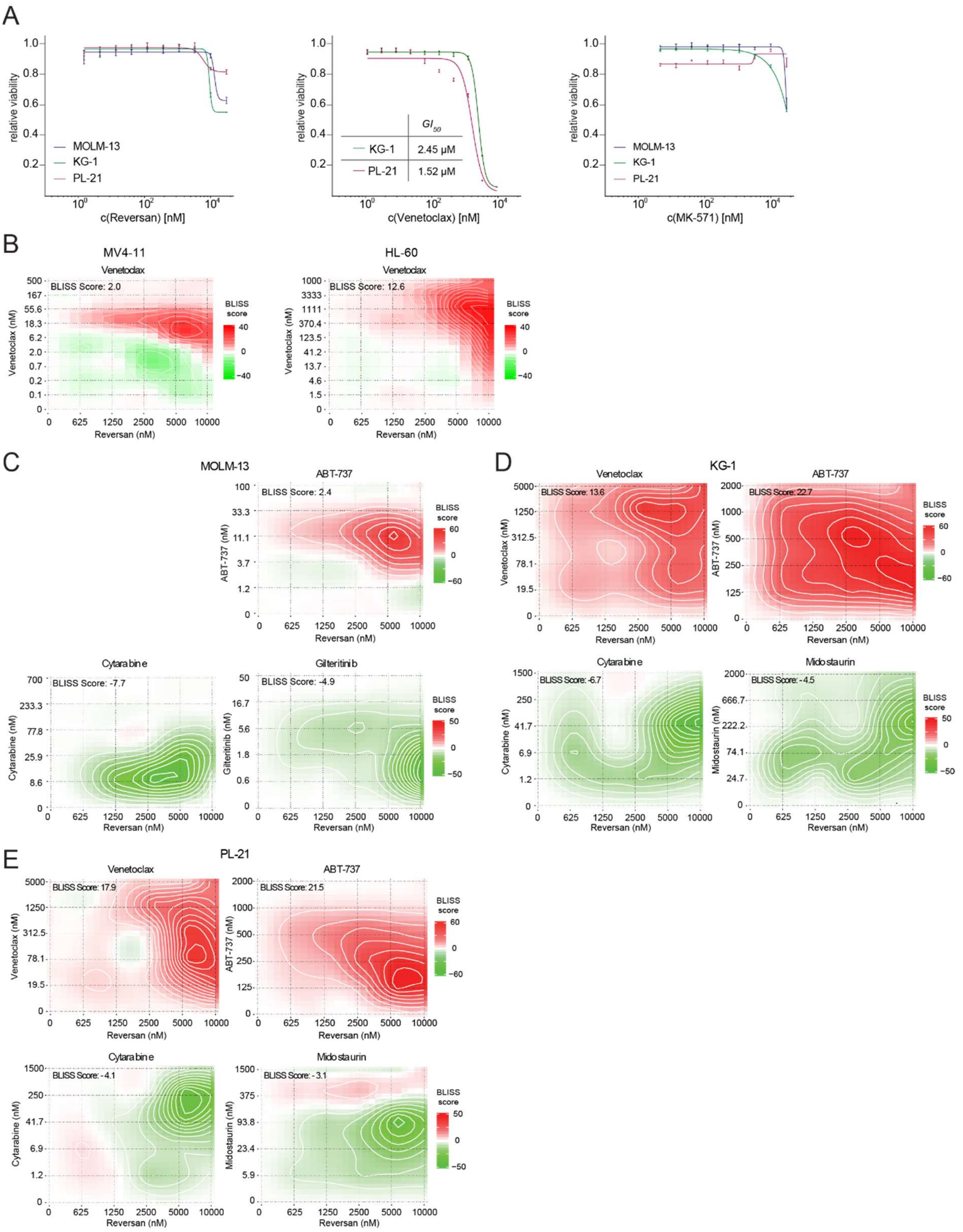

Supplementary Figure 2: Pharmacological inhibition of ABCC1 synergizes with BCL-2-inhibitor treatment

(A) 5-day dose-response curves of MOLM-13, KG-1 and PL-21 cells treated with Reversan (top left) or MK-571 (bottom) treatment or of KG-1 and PL-21 cells upon Venetoclax treatment (top right). Data are presented as mean values ± SD. n=3 experimental replicates. (B-E) BLISS synergy score distribution of AML cell lines treated with indicated drugs at indicated

concentrations in combination with Reversan for 5 days, as determined by SynergyFinder. (B) MV4-11 and HL-60 cells. (C) MOLM-13 cells. (D) KG-1 cells. (E) PL-21 cells. (A-E) Source data are provided as a Source Data file.

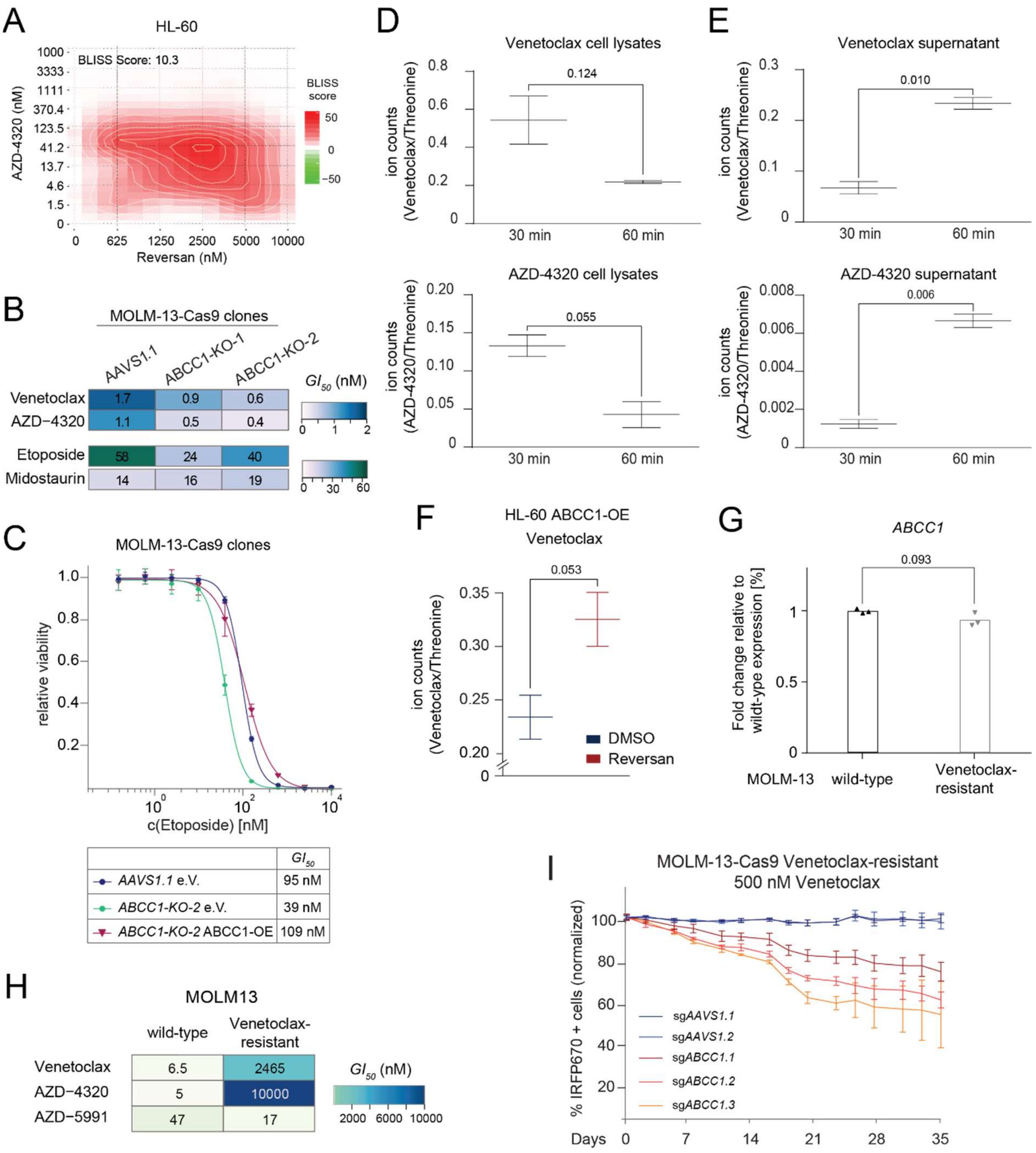

Supplementary Figure 3: Validation of MOLM-13-Cas9 knockout clones and Venetoclax-resistant MOLM-13-Cas9 cells

(A) BLISS synergy score distribution of HL-60 cells treated with AZD-4320 at indicated concentrations in combination with Reversan for 5 days, as determined by SynergyFinder. (B) Heatmap of GI_{50} values [nM] of 5-day viability assays with indicated drugs in MOLM-13-Cas9 knockout clones (*AAVS1.1, ABCC1-KO-1* and *ABCC1-KO-2*). n=3 experimental replicates. (C) Dose-response curves of Etoposide in MOLM-13-Cas9 *AAVS1.1* or *ABCC1-KO-2 clones* transduced with either an empty vector (e.V.) or an ABCC1-overexpressing (OE) construct after 5 days of treatment. Data are presented as mean values ± SD. n=3 experimental replicates. (D-E) Ion count ratios of Venetoclax (top panels) or AZD-4320 (bottom panels) to

Threonine (control amino acid) of ABCC1-overexpressing HL-60 cells, as determined by LC-MS/MS. Cells were treated with the respective drug for 30 and 60 min, washed and the culture media was replaced. Data are presented as mean values ± SD. n=2 experimental replicates. Significance was determined with an unpaired Student's t-test with two-tailed p-value as indicated. (D) Ion count detection in cell lysates. (E) Ion count detection in culture supernatants. (F) Ion count ratio of Venetoclax to Threonine (control amino acid) of ABCC1-overexpressing HL-60 cells upon co-treatment of either DMSO or Reversan (10 µM) as determined by LC-MS/MS. Data are presented as mean values ± SD. n=2 experimental replicates. Significance was determined with an unpaired Student's t-test with two-tailed p-value as indicated. (G) RT-qPCR analysis of *ABCC1* mRNA expression in wild-type (WT) and Venetoclax-resistant MOLM-13 cells. Expression was normalized to *ACTB* and to MOLM-13 WT cells. Data are presented as mean values. n=3 technical replicates, n=1 experimental replicate. Significance was determined with an unpaired Student's t-test with two-tailed p-value as indicated. (H) Heatmap of GI_{50} values [nM] of 5-day viability assays with indicated drugs in MOLM-13 WT and Venetoclax-resistant cells. n=3 experimental replicates. (I) Competitive proliferation assay of Venetoclax-resistant Cas9-expressing MOLM-13 cells transduced with indicated sgRNAs upon Venetoclax (500 nM) treatment for 38 days. Percentages of sgRNA/IRFP670+ cells were normalized to day 0 of treatment and to DMSO controls. Data are presented as mean values ± SD. n=3 experimental replicates. (A-I) Source data are provided as a Source Data file.

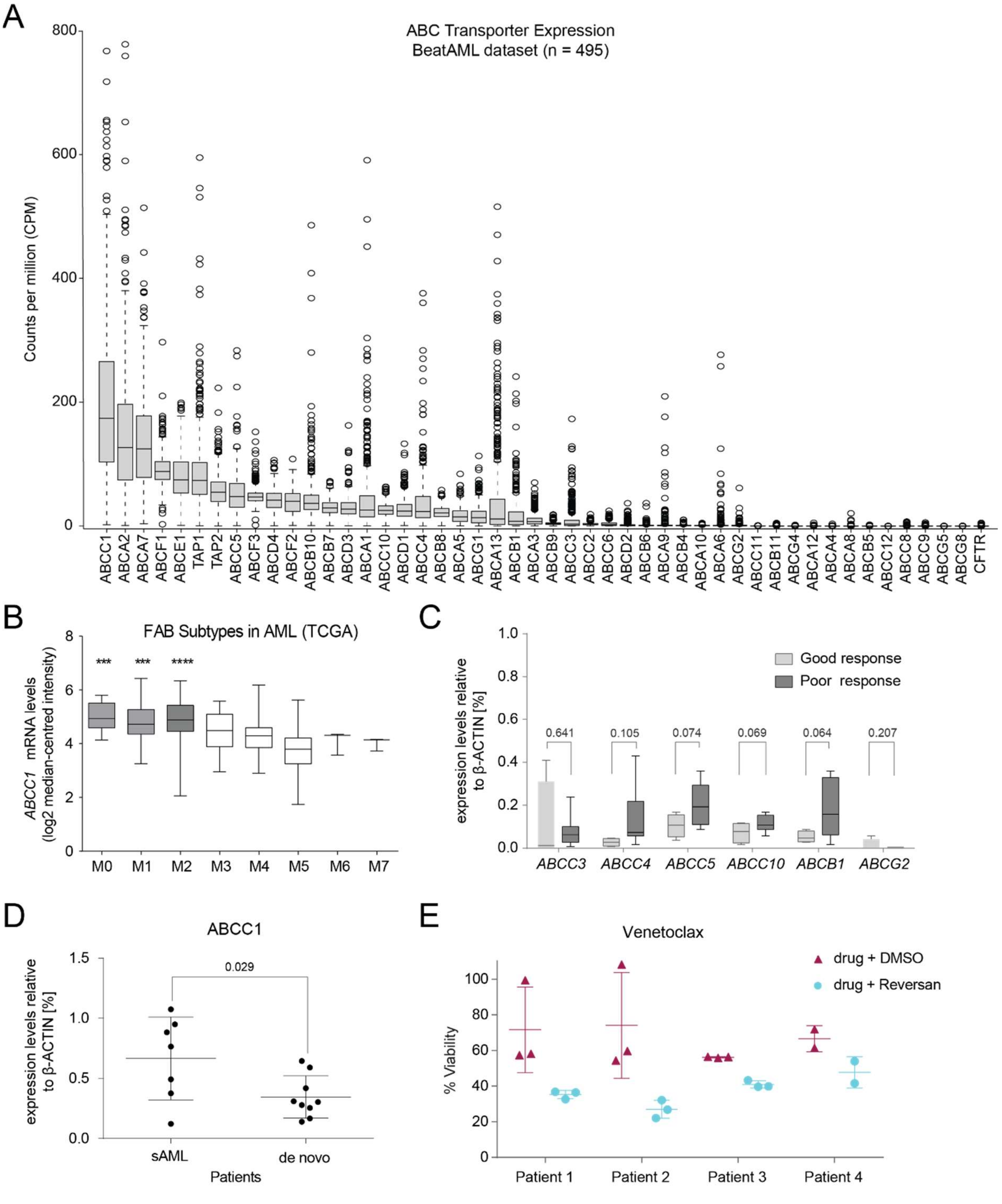

Supplementary Figure 4: ABCC1 levels dictate the response of AML to Venetoclax

(A) *ABC* transporter mRNA expression levels (counts per million (CPM)) in AML patient samples (n=495). Data were extracted from the BeatAML dataset. Boxes represent interquartile ranges; horizontal lines represent the median, whiskers indicate ± SD. (B) *ABCC1* mRNA expression levels in AML patient samples (n=197) grouped according to the FAB classifications. Data and statistical analysis were extracted from the Oncomine Platform, from the TCGA Leukemia dataset. Boxes represent interquartile range, horizontal lines represent the mean, whiskers indicate lower and upper limits. The FAB classification groups were

compared to each other for significance ***p<0.001, ****p<0.0001. (C) Comparison of *ABCC3, ABCC4, ABCC5, ABCC10, ABCB1* and *ABCG2* expression levels between patients with good or poor response to Venetoclax treatment (n=14 individual patient samples – same as in (6C) – 2 non-responders excluded). Boxes represent interquartile ranges; horizontal lines represent the median expression; whiskers indicate lower and upper limits of the respective patient cohort. Analysis of expression levels of each patient sample was performed in duplicates. Significance was determined with an unpaired Student's t-test with two-tailed p-value as indicated. (D) Comparison of *ABCC1* expression levels between patients with secondary AML (sAML) and de novo AML. n=16 individual patient samples (same as in 6C). Analysis of expression levels of each patient sample was performed in technical duplicates, relative to ACTB [%]. Data are presented as mean values ± SD. Significance was determined with an unpaired Student's t-test with two-tailed p-value as indicated. (E) Relative viability of primary patient-derived AML cells (patient 1-4, same as in (6E)) treated with Venetoclax in combination with either DMSO or Reversan for 3 days. Selected concentrations depicted were: patient 1: 39 nM Venetoclax and 5 µM Reversan; patient 2: 2.5 µM Venetoclax and 5 µM Reversan; patient 3: 156.3 nM Venetoclax and 2.5 µM Reversan; patient 4: 39 nM Venetoclax and 2.5 µM Reversan. Data is normalized to DMSO-treated control. Data are presented as mean values ± SD. n=2 (patient 4)/ n=3 (patient 1-3) experimental replicates of the same patient. Additional information on the mutational status of the patients is provided in Supplemental Table S1. (C-E) Source data are provided as a Source Data file.

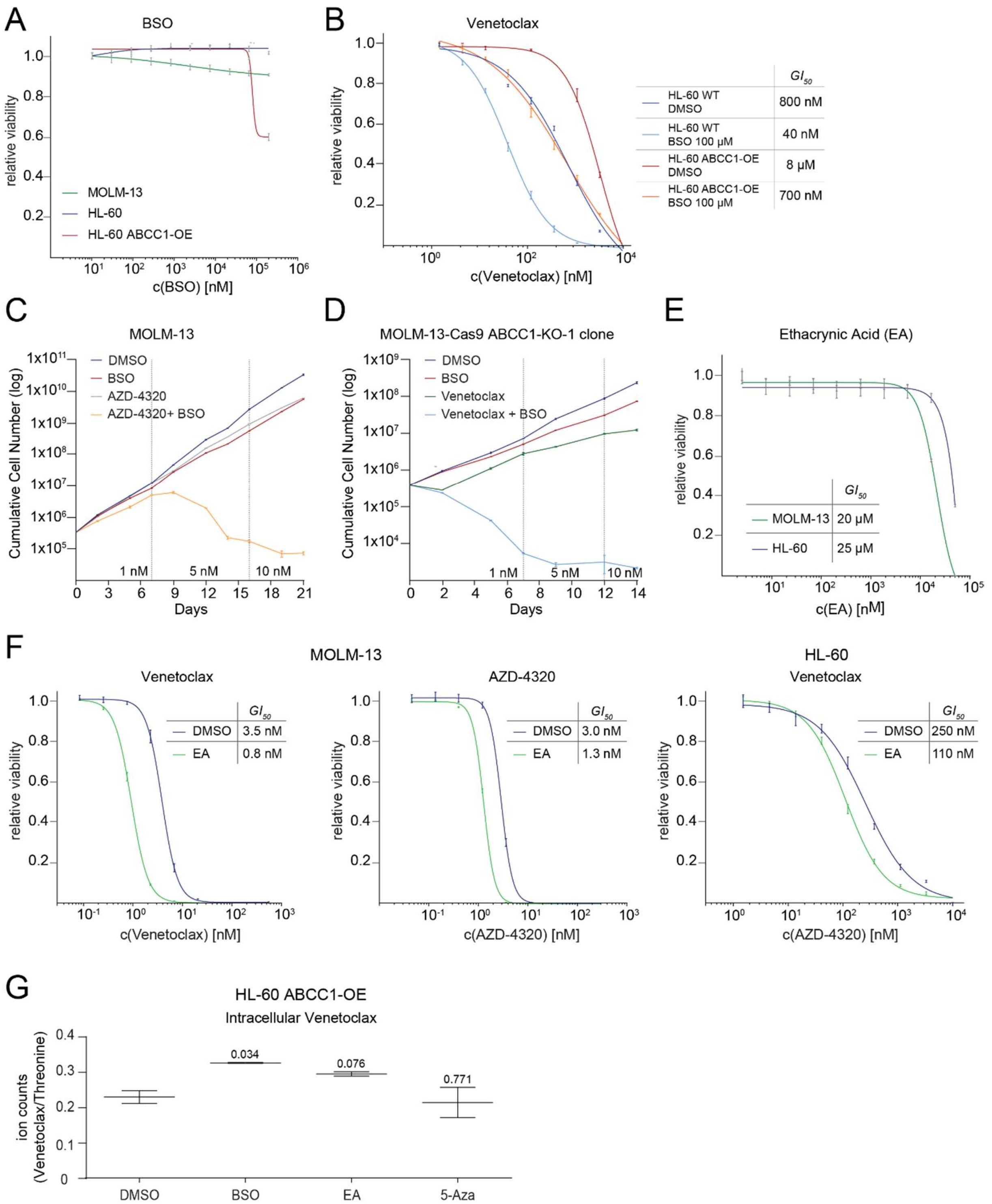

Supplementary Figure 5: Glutathione metabolism modulates the sensitivity to BH3 mimetics

(A) Dose-response curves of Buthionine sulfoximine (BSO) in MOLM-13, HL-60 wild-type (WT) and HL-60 ABCC1-overexpressing (OE) cells after 5 days of treatment. Data are presented as mean values ± SD. n=3 experimental replicates. (B) Dose-response curves of Venetoclax in HL-60 WT and ABCC1-OE cells co-treated with either DMSO or 100 µM BSO for 5 days. Data are presented as mean values ± SD. n=3 experimental replicates. (C) Growth curves of MOLM-

13 cells treated with DMSO, 100 µM BSO, AZD-4320 (increasing concentrations 1, 5 and 10 nM) or AZD-4320 in combination with 100 µM BSO. Data are presented as mean values ± SD. n=2 experimental replicates. (D) Growth curves of MOLM-13-Cas9 *ABCC1-KO-1* clone treated with DMSO, 100 µM BSO, Venetoclax (increasing concentrations 1, 5 and 10 nM) or Venetoclax in combination with 100 µM BSO. Data are presented as mean values ± SD. n=2 experimental replicates. (E) Dose-response curves of Ethacrynic acid (EA) in MOLM-13 and HL-60 cells after 5 days of treatment. Data are presented as mean values ± SD. n=3 experimental replicates. (F) Dose-response curves of Venetoclax (left) or AZD-4320 (middle) in MOLM-13 and AZD-4320 treatment in HL-60 cells (right), co-treated with either DMSO or 10 µM EA for 5 days. Data are presented as mean values ± SD. n=3 experimental replicates. (G) Ion count ratio of intracellular Venetoclax to Threonine (control amino acid) of ABCC1-overexpressing HL-60 cells upon co-treatment with either DMSO, BSO (100 µM), Ethacrynic acid (EA, 10 µM) or 5-Azacitidine (5-Aza, 5 µM) as determined by LC-MS/MS. n=2 experimental replicates. Data are presented as mean values ± SD. Significance was determined with an unpaired Student's t-test with two-tailed p-value as indicated. (A-G) Source data are provided as a Source Data file.

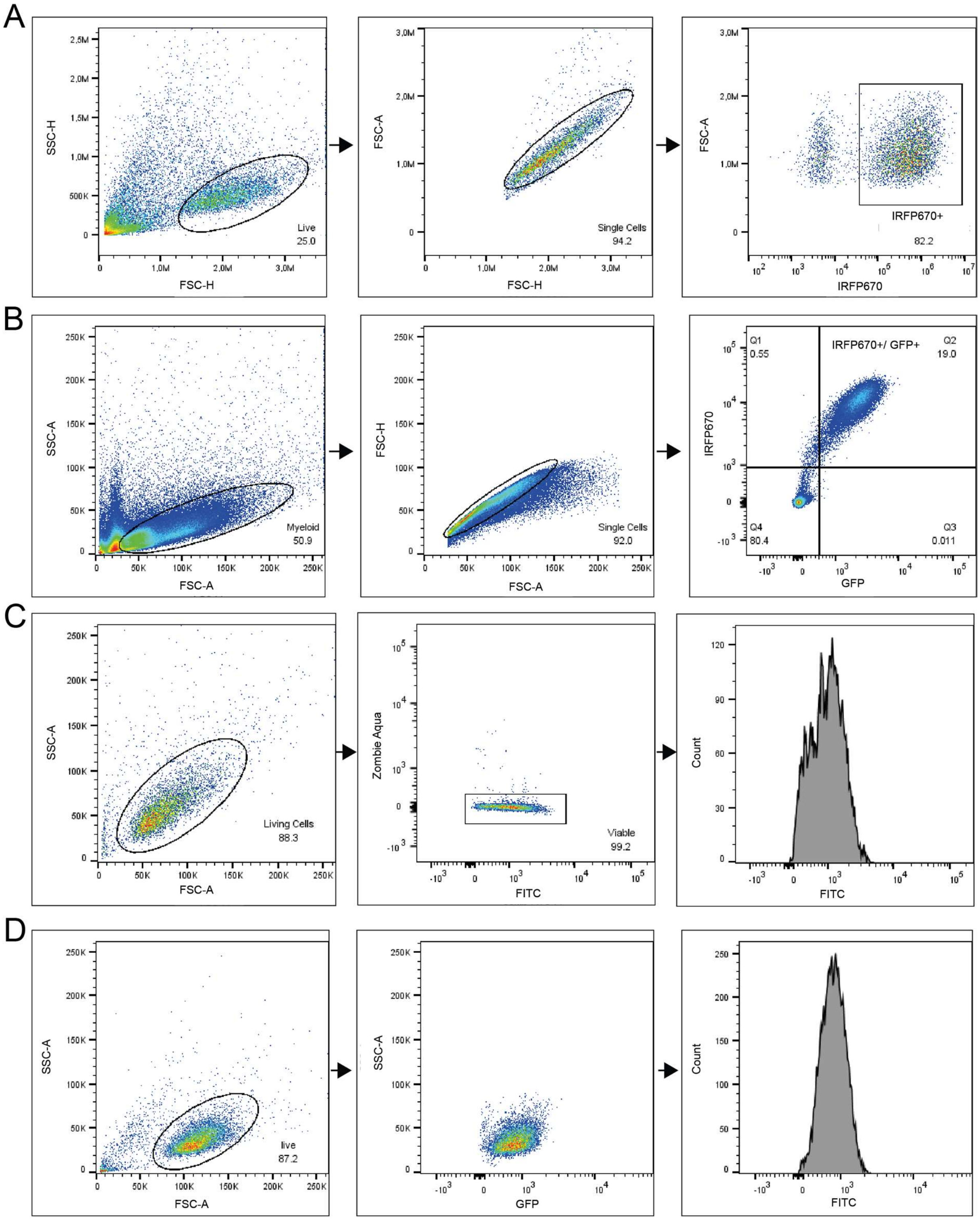

Supplementary Figure 6: Flow Cytometry Gating Strategies

(A) Competitive cell proliferation assays were measured on an IntelliCyt IQueScreener Plus to monitor transduced IRFP670$^+$ sgRNA-expressing cells over time. Corresponding Figures: 1A-C; 3B-C; 5B; S1A,D,E; S3 (B) Quantification of IRFP670$^+$ human cells in the mouse bone marrow on a FACS Canto II flow cytometer. Corresponding Figure: 3F (C) Intracellular staining of ABCC1 (ab24102, Abcam) quantified on a FACS Canto II flow cytometer. Cell samples were stained with Zombi Aqua^{TM} viability dye (BioLegend, 423113), and gated on the Zombi Aqua

negative population. The ABCC1 stained cells were stained using an anti-Mouse FITC-labelled secondary antibody (Alexa Fluor™ 488, A-21202, Thermo Fischer Scientific). Corresponding Figure: 4B (D) Quantification of the fluorescent product in the Calcein-acetoxymethyl ester (Calcein-AM) transport assay, measured in the FITC channel of a FACS Canto II flow cytometer. Corresponding Figures: 4G-H

FSC- forward-scatter; SSC- Side Scatter; H- height; A- area; IRFP- near-infrared fluorescent protein; GFP- green fluorescent protein; FITC- fluorescein isothiocyanate

Supplementary Table 1: Patient Data

RNA samples - qPCR/ mRNA expression analysis

sample	*sex*	*FAB classification*	*mutations (NGS & PCR)*	*treatment response*	*date of sample*	*start Venetoclax treatment*	*previous therapy*
#1	f	M1	-	cytoreduction	2016-10-10	2017-07-14	chemotherapy
#2	m	M4	-	cytoreduction	2017-06-30	2017-07-19	5-Azacytidine
#3	m	sAML	CBL, IDH1, RUNX1 (11.2.19)	cytoreduction	2019-02-12	2019-02-20	5-Azacytidine
#4	m	sAML	n.a.	cytoreduction	2017-11-03	2018-04-10	5-Azacytidine
#5	m	sAML	n.a.	stable disease	2018-12-14	2019-01-25	chemotherapy, 5-Azacytidine
#6	f	sAML	CALR (19.9.17)	cytoreduction	2017-10-03	2017-10-03	5-Azacytidine
#7	m	RAEB	-	cytoreduction	2019-01-25	2019-01-29	5-Azacytidine
#8	m	AUL	-	complete remission with incomplete hematologic recovery (Cri)	2018-12-20	2019-01-03	chemotherapy
#9	m	M4	KRAS, SF3B1 (6.11.18)	stable disease	2018-11-06	2019-01-08	chemotherapy
#10	m	sAML	-	stable disease	2019-02-20	2019-03-06	no
#11	m	sAML	ASXL1, CBPA, NRAS, RUNX1, SRSF2, TET2 (12.11.19)	complete remission with incomplete hematologic recovery (Cri)	2019-11-12	2019-11-21	5-Azacytidine
#12	m	sAML	TP53 (29.10.19)	non-responder*	2019-05-23	2019-11-23	chemotherapy
#13	f	M4	NPM1, ASXL1, IDH2, SRSF2 (3.9.2019)	complete remission (CR)	2019-09-03	2020-01-03	chemotherapy, 5-Azacytidine
#14	f	-	CALR, SF3B1 (24.1.2020)	cytoreduction	2020-01-24	2020-02-03	no
#15	f	-	DNMT3A, TET2 (11.12.2019)	complete remission (CR)	2020-02-05	2020-02-06	chemotherapy
#16	f	M0	FLT3 ITD pos., NPM1, ASXL1, CSF3R, TET2 (27.2.18)	non-responder*	2020-10-07	2020-11-23	chemotherapy

* Non-responders were excluded from the analysis in Figure 6D and Supplementary Figure 4C

AML cells in culture for viability assays

sample	*sex*	*FAB classification*	*mutations (NGS & PCR)*	*% blasts in bone marrow*

patient 1	m	M2	CEBPA, DNMT3A, EZH2, U2AF1 (18.11.20)	75
patient 2	f	M4	FLT3 ITD pos. (2.3.17)	76
patient 3	m	M1	-	90
patient 4	m	M1	DNMT3A, WT1 (7.10.19)	85

Supplementary Table 2: sgRNA, qPCR, genotyping – Oligos/ Primers used

**sgRNA human**		**sgRNA human**	
target	***sgRNA sequence***	***target***	***sgRNA sequence***
AAVS1_1	GCTCCGGAAAGAGCATCCT	*ABCC2_1*	GAGAGCTGCAGAAAGCCAGG
AAVS1_2	GCTGTGCCCCGATGCACAC	*ABCC2_2*	GATTGGTATATCGAACAGCA
RPL17_1	GTACCATTCCGACGTTACAA	*ABCC3_1*	GCTCATACTGTATCAGCA
RPL17_2	GACATCTTTCAGATACTTCG	*ABCC3_2*	GGCTAAAAGGATCTTGGAG
ABCA1_1	GCCTGTTCTCAGATGCTCGG	*ABCC4_1*	GGAAGTTGTTCTCATCACTG
ABCA1_2	GAGTCGGGTAACGGAAACAG	*ABCC4_2*	GAAGAACACGCGTGAGCAG
ABCA10_1	GCCAGTATGAAATACAATT	*ABCC5_1*	GGATGAGGAGCATCCCAA
ABCA10_2	GGCAAAGTACTGGCTAAAA	*ABCC5_2*	GTGATCATCAGGCACACGA
ABCA12_1	GATAATATAACGCATGTG	*ABCC6_1*	GATGGGACCAAGGACCCAG
ABCA12_2	GGTGTCATCCTGAACCGGGG	*ABCC6_2*	GTGCTTCCTGAGAACAGCAG
ABCA13_1	GGATCTCAATAAGACCGAGG	*ABCC8_1*	GTTGTGATAGTAGACCACGG
ABCA13_2	GAGCCAATTCTTCCACAGCA	*ABCC8_2*	GATGACATTGACCTCCACG
ABCA2_1	GCAACCTGTTTGACCCAGCG	*ABCC9_1*	GTGGCAACGAATCCCATCA
ABCA2_2	GGATAAAGAACAGCACCA	*ABCC9_2*	GTCAAAAGCAACATACTGG
ABCA2_3	GGGCAATCCCCTGTTCCGGA	*ABCD1_1*	GGCCGTGAGGAACACCACG
ABCA2_4	GCAATCGCTGTGCCCGGA	*ABCD1_2*	GGCCACATACACCGACAGGA
ABCA3_1	GCACCCCTTCAACCACAGCA	*ABCD2_1*	GTGACCACTGAAACAGGG
ABCA3_2	GAGCAGGGTCATGAAGGAGG	*ABCD2_2*	GTGGCTCTGCCTGCACTCAG
ABCA4_1	GAAGGACATCGCCTGCAGCG	*ABCD3_1*	GCTCACTAAATACCTCTATG
ABCA4_2	GATGATGAAGCGCTCCAGGA	*ABCD3_2*	GATGTTTGGATGATTCAAAA
ABCA5_1	GAAATAGATACCTTTCCCCG	*ABCD4_1*	GAGACTCCTTCAGACCCAGA
ABCA5_2	GTAAAACTGTGAAACCTG	*ABCD4_2*	GTAGGGTCAGGCACAAAA
ABCA6_1	GAGAAATCTTCTTTCCAAAG	*ABCE1_1*	GACATCTAGGTAACTAGA
ABCA6_2	GGCTCCTCAGAATCTGGGAA	*ABCE1_2*	GCCTTTTAGTGTAAGAGA

ABCA7_1	GTGCAAAGAGTAGCTTCCCG	*ABCF1_1*	GACTTCTCCGTGTCCCAGG
ABCA7_2	GCAGGGTGAGCTCCTCGA	*ABCF1_2*	GGAGCCTCCCAAACAAGGGA
ABCA8_1	GTTAATGTCACAAGAGAG	*ABCF2_1*	GTCCATGCAAGATCCGCG
ABCA8_2	GACCCATCATTGTCATCA	*ABCF2_2*	GCTGGAGAAAGAGGCAGAG
ABCA9_1	GGCCATAGAGGAGAAAGA	*ABCF3_1*	GAATGCAAAGAAGTTAGAGA
ABCA9_2	GCTTCAGCCCCATTCCTAAA	*ABCF3_2*	GCCAGGCAGGCAGCAGAA
ABCB1_1	GAGAGGAAGTCCAGCCCCA	*ABCG1_1*	GGAGAAGGATGAAGGCAGAA
ABCB1_2	GGTATGCCTATTATTACAG	*ABCG1_2*	GCCAGGATGTTCATCAGCG
ABCB10_1	GTGCAGTGTCTGATGAGAGG	*ABCG2_1*	GGCTCATCCAAGAACAAGA
ABCB10_2	GTCGCTGTAGTCCACAGTG	*ABCG2_2*	GCTGCAAGGAAAGATCCAAG
ABCB11_1	GAAGGCCTATGCCAAAGCAG	*ABCG4_1*	GATGCCAATCAGCTCCCGG
ABCB11_2	GCTCCAATCCCAATGAGA	*ABCG4_2*	GGGTTGTTGACCAGCTCCA
ABCB4_1	GATGCATATATTAACAGGA	*ABCG5_1*	GCGAGCCAGTTCCACCAGG
ABCB4_2	GCTGATGGCCATTATCACAA	*ABCG5_2*	GCTCTCCGAAGCTCAGGA
ABCB5_1	GCTCTTCACAGAGTGCAG	*ABCG8_1*	GTGAAGCTGTCGAGCCCAG
ABCB5_2	GCAGCACGACAGACCAAG	*ABCG8_2*	GTGATCACATCTAGCAAGG
ABCB6_1	GCAGGGAAGAGCCAGCACCA	*CFTR_1*	GTCAGCAGAATCAACAGAA
ABCB6_2	GGGCTGTTCCAAGACACCA	*CFTR_2*	GAGGAGTGCCACTTGCAA
ABCB7_1	GTTGATGTGGCCTCCACTG	*TAP1_1*	GTAGAATCCAGTCAGTGAGG
ABCB7_2	GGCATGGTCATGCAGGAGG	*TAP1_2*	GGGATCTATAACAACACCA
ABCB8_1	GGTCGTGGCCAAGTACACAA	*TAP2_1*	GGACAAGGAAGAAGAAGG
ABCB8_2	GCAGCACCAGGTACCCGA	*TAP2_2*	GCCAGTTACTCATCAGGG
ABCB9_1	GACGATGCCATCAATGGCG		
ABCB9_2	GAGGGTGATGACCAGCCACG	**sgRNA mouse**	
ABCC10_1	GGGAAGACGGAAAGCAGGA	***target***	***sgRNA sequence***
ABCC10_2	GGAATGTGCCAACACCCACA	*sgRosa.26*	GAAGATGGGCGGGAGTCTTC
ABCC1_1	GTATAACACCTTAAACAGAG	*sgMyb.33*	GAAGCTGGTGGAACAGAA
ABCC1_2	GGATGGTTTCCGAGAACAG	*sgAbcc1_1*	GGGCTGACCAGAAACACTG
ABCC1_3	GGGCTGACCAGAAACACTG	*sgAbcc1_2*	GAAGGGGAAACAGGCCCAG
ABCC1_4	GAAGGTGATCCTCGACAGGA	*sgAbcc1_3*	GAAGGTGATCCTCGACAGGA
ABCC11_1	GGGTGAGCCATGACACGGTG		
ABCC11_2	GAGACTTCTTCTTCCCAA		
ABCC12_1	GGGCCATCAACTACCGCA		
ABCC12_2	GCCAACCCAGACTTTCCCAG		
**qPCR (mRNA expression)**			
Target	***Forward primer***	***Reverse primer***	
β-ACTIN	GCTCATAGCTCTTCTCCAGGG	CCTGAACCCTAAGGCCAACCG	

ABCB1	GGAAAGTGCTGCTTGATGGC	AGGCATGTATGTTGGCCTCC
ABCG2	CATGGTGTATAGACGCCCTGAC	GTTCCCAAATTGATGTTGTGACAGA
ABCC1	TTACTCATTCAGCTCGTCTTGTC	CAGGGATTAGGGTCGTGGAT
ABCC2	TCCTTTGCAAGTGACCGTGA	CCTTCCTGGCCAAGTTGGAT
ABCC3	CCTTTGCCAACTTTCTCTGC	AGGGCACTCAGCTGTCTCAT
ABCC4	CATGACTTGGACACGGTAACTG TTG	TCAGGAATGTCGGTTAGAGGTTTG
ABCC5	GGAGCTCTCAATGGAAGACG	CACACGATGGACAGGATGAG
ABCC6	CACAGTTTGTGCTGTCCTGC	CCAAGCGACCAGAGGTCTTT
ABCC10	AAACCAGAGGTGCCAGTTTG	TGGCCTCTGTCTGTGTGAAG
**genotyping**		
Target	***Forward primer***	***Reverse primer***
sgABCC1.1 target region	CCCTGAAGGGTGACATTCCC	CTGCTGGCATTTCCTTGCTC
sgABCC1.2 target region	GCCGGCTTTCTGCCATTACA	CTGCAAGACAGCAGTTCATCAAT
