# Supplementary material for: ABCC1 and glutathione metabolism limit the efficacy of BCL-2 inhibitors in acute myeloid leukemia
Source: Nat Commun. 2023 Sep 19;14:5709. doi: 10.1038/s41467-023-41229-2 (PMC10509209; doi:10.1038/s41467-023-41229-2)
Supplement: Supplementary file 3 — Reporting Summary [file 41467_2023_41229_MOESM3_ESM.pdf]

nature portfolio

Corresponding author(s): Florian Grebien

Last updated by author(s): Jul 21, 2023

Reporting Summary

Nature Portfolio wishes to improve the reproducibility of the work that we publish. This form provides structure for consistency and transparency in reporting. For further information on Nature Portfolio policies, see our Editorial Policies and the Editorial Policy Checklist.

Statistics

For all statistical analyses, confirm that the following items are present in the figure legend, table legend, main text, or Methods section.

n/a	Confirmed	
☐	☒	The exact sample size (n) for each experimental group/condition, given as a discrete number and unit of measurement
☐	☒	A statement on whether measurements were taken from distinct samples or whether the same sample was measured repeatedly
☐	☒	The statistical test(s) used AND whether they are one- or two-sided<br>*Only common tests should be described solely by name; describe more complex techniques in the Methods section.*
☒	☐	A description of all covariates tested
☐	☒	A description of any assumptions or corrections, such as tests of normality and adjustment for multiple comparisons
☐	☒	A full description of the statistical parameters including central tendency (e.g. means) or other basic estimates (e.g. regression coefficient) AND variation (e.g. standard deviation) or associated estimates of uncertainty (e.g. confidence intervals)
☐	☒	For null hypothesis testing, the test statistic (e.g. F, t, r) with confidence intervals, effect sizes, degrees of freedom and P value noted<br>*Give P values as exact values whenever suitable.*
☒	☐	For Bayesian analysis, information on the choice of priors and Markov chain Monte Carlo settings
☒	☐	For hierarchical and complex designs, identification of the appropriate level for tests and full reporting of outcomes
☐	☒	Estimates of effect sizes (e.g. Cohen's d, Pearson's r), indicating how they were calculated

Our web collection on statistics for biologists contains articles on many of the points above.

Software and code

Policy information about availability of computer code

Data collection	Flow cytometry: Stained samples were measured using a FACS Canto II flow cytometer (BD Biosciences). Competition assay and growth curve was measured using IntelliCyt IQueScreener Plus (BioScience, Sartorius Group, Germany) with the Software Forecyt standard Edition 7.0 (R2) (7.0.7035).<br><br>Cell viability: Determined using the CellTiter-Glo® Luminescent Cell Viability Assay (Promega, USA), on a Spark multimode microplate reader (TECAN, Switzerland).<br><br>Genotyping PCR analyses: PCR products were purified and analyzed by Sanger Sequencing. Tracking of Indels by Decomposition (TIDE)was used to identify the type of insertions and/or deletions (TIDE version 3.3.0).<br><br>Whole-body fluorescence imaging was done using the IVIS optical imaging system (PerkinElmer, USmA). Signal quantification was performed using the Living Image analysis software (PerkinElmer, USA). Living image analysis software Version 4.7.3.<br><br>Real-time PCR analysis were performed on a Bio-Rad CFX96-Real-Time PCR Detection System.<br><br>LC-MS/MS analysis was performed with an Ultimate 3000 HPLC system (Thermo Fisher Scientific, Dreieich, Germany) coupled to a Thermo Q Exactive Focus mass spectrometer (Thermo Fisher Scientific)
Data analysis	Flow cytometry analysis: FlowJo software v10.8.1(FlowJo, LLC). Forecyt standard Edition 7.0 (R2) (7.0.7035).<br>LC-MS/MS: Data analysis was performed with Compound Discoverer Version 3.0 (Thermo Fisher Scientific) and data interpretation was

performed using TraceFinder Version 5.0 (Thermo Fisher Scientific)
The Prism 6.0.1 software (Graphpad, USA) and Microsoft Excel 2016 were used for data compilation and for statistical analyses.
BLISS score was calculated using the SynergyFinder online tool, Version 3.

For manuscripts utilizing custom algorithms or software that are central to the research but not yet described in published literature, software must be made available to editors and reviewers. We strongly encourage code deposition in a community repository (e.g. GitHub). See the Nature Portfolio guidelines for submitting code & software for further information.

Data

Policy information about availability of data

All manuscripts must include a data availability statement. This statement should provide the following information, where applicable:

- Accession codes, unique identifiers, or web links for publicly available datasets
- A description of any restrictions on data availability
- For clinical datasets or third party data, please ensure that the statement adheres to our policy

All data generated in this study are provided in the Source Data file. Publicly available datasets used are not in the Source Data file, but is listed. Gene expression data for ABCC1 were extracted from the Haferlach Leukemia, Valk Leukemia or TCGA Leukemia datasets (reporter: 202804_at) using the Oncomine™ Research Premium Edition database (Thermo Fisher, USA) (88), accessed in July, 2021. Gene expression analysis of ABC transporters in AML patients in the BeatAML dataset (47) was accessed in January, 2021 from the NCI Genomic Data Commons: https://gdc.cancer.gov/about-data/publications/BEATAML1-0-COHORT-2018. The Ordino database was used to extract gene expression data in human AML cell lines (KG-1, THP-1, PL-21, MV4-11, HL-60, MOLM-13). The Ordino database contains data from: The Cancer Genome Atlas (TCGA), the Cancer Cell Line Encyclopedia (CCLE), two depletion screen data sets (89,90); data extracted from August, 2021.

Research involving human participants, their data, or biological material

Policy information about studies with human participants or human data. See also policy information about sex, gender (identity/presentation), and sexual orientation and race, ethnicity and racism.

Reporting on sex and gender	We did not distinguish between sex in our analysis, but we made sure to include both female and male patients, based on availability.
Reporting on race, ethnicity, or other socially relevant groupings	not applicable
Population characteristics	Primary AML samples were obtained by bone marrow puncture or venipuncture during routine investigations at the time of diagnosis. Cells were stored in a local biobank until used. All patients gave written informed consent before bone marrow or blood was obtained.
Recruitment	Samples of AML patients prior to Venetoclax treatment were selected to evaluate if ABC transporter expression is predictive of treatment response. Venetoclax treatment response was evaluated by the medical doctors based on the patient's remission.
Ethics oversight	Primary AML samples were obtained by bone marrow puncture or venipuncture during routine investigations at the time of diagnosis. Cells were stored in a local biobank until used. All patients gave written informed consent before bone marrow or blood was obtained. The study was approved by the ethics committee of the Medical University of Vienna (EK-No: 1355/2021).

Note that full information on the approval of the study protocol must also be provided in the manuscript.

Field-specific reporting

Please select the one below that is the best fit for your research. If you are not sure, read the appropriate sections before making your selection.

☒ Life sciences ☐ Behavioural & social sciences ☐ Ecological, evolutionary & environmental sciences

For a reference copy of the document with all sections, see nature.com/documents/nr-reporting-summary-flat.pdf

Life sciences study design

All studies must disclose on these points even when the disclosure is negative.

Sample size	An appropriate sample size was chosen based on the magnitude and consistency of measurable differences between groups. All sample sizes are indicated in the figure legends. Sample was choosen based on availability and practical reasons and costs.
Data exclusions	Non-responders were excluded from the analysis in Figure 6C and Suppl. Figure 4C.
Replication	Numbers of biological and/or technical replicates are indicated in the figure legends.
Randomization	Age-matched mice (16-20 weeks old) were randomly distributed into two groups of equal size and transplanted with equal cell numbers of either MOLM-13 Cas9 AAVS1.1 or ABCC1 KO cells. Both cohorts were treated with 10 mg/kg AZD-4320 or vehicle.<br>Cell culture experiments were not randomized as the experimental groups needed to be known for subsequent analysis.

Blinding	Investigators were not blinded to the group allocation of mice since the experimental setup defined an equal endpoint for both groups and the kowledge of the genotype of transplanted cells needed to be known for subsequent analysis.

Reporting for specific materials, systems and methods

We require information from authors about some types of materials, experimental systems and methods used in many studies. Here, indicate whether each material, system or method listed is relevant to your study. If you are not sure if a list item applies to your research, read the appropriate section before selecting a response.

Materials & experimental systems

n/a	Involved in the study
☐	☒ Antibodies
☐	☒ Eukaryotic cell lines
☒	☐ Palaeontology and archaeology
☐	☒ Animals and other organisms
☒	☐ Clinical data
☒	☐ Dual use research of concern
☒	☐ Plants

Methods

n/a	Involved in the study
☒	☐ ChIP-seq
☐	☒ Flow cytometry
☒	☐ MRI-based neuroimaging

Antibodies

Antibodies used	Anti MRP1 ab24102, lot: GR284248-19, Dilution 1:200<br>Goat anti Mouse igG Catalogue Number: P31582, lot: 1915874, Dilution 1:200
Validation	anti-ABCC1 antibody was validated in cells ectopically overexpressing ABCC1 compared to WT cells. It was validated in intracellular FACS staining as well as in WB. It has been published 56 times according to Abcam, most recently: Hanssen KM et al. GSH facilitates the binding and inhibitory activity of novel multidrug resistance protein 1 (MRP1) modulators. FEBS J 289:3854-3875 (2022).

Eukaryotic cell lines

Policy information about cell lines and Sex and Gender in Research

Cell line source(s)	All human AML cell lines, HL-60 (ACC 3), MOLM-13 (ACC 554), MV4-11(ACC 102), THP-1 (ACC 16), KG-1 (ACC 14), PL-21 (ACC 536) were purchased from DSMZ.
Authentication	Cell lines were fingerprinted using STR analysis.
Mycoplasma contamination	All cell lines were routinely tested for mycoplasma contamination and confirmed negative.
Commonly misidentified lines<br>(See ICLAC register)	No commonly misidentified cell lines were used.

Animals and other research organisms

Policy information about studies involving animals; ARRIVE guidelines recommended for reporting animal research, and Sex and Gender in Research

Laboratory animals	NOD.Cg-Prkdcscid Il2rgtm1Wjl Tg(CMV-IL3,CSF2,KITLG)1Eav/MloySzJ mice expressing human IL-3, GM-CSF and SCF on a NSG background (NSG-S mice) were purchased from Jackson Laboratory (Bar Harbor, ME, USA). NSG-S mice were kept in specific opportunistic pathogen free quality (SOPF) under stringent controlled standard conditions, in individually ventilated cages, fed with SSNIFF Haltungsfutter (CHOW standard 10mm pellets; Catalog-No. V1534-000), ad libitum. Mice were 16-20 weeks old, 8 males and 11 females - mixed in the groups
Wild animals	No wild animals were used in the study.
Reporting on sex	Male and female mice were distributed in the different groups.<br>We didn't distinguish between the sex in our study because according to the literature we didn't expect any difference and also could not observe any differenceb in leukemia onset or treatment response.
Field-collected samples	No field collected samples were used in the study.
Ethics oversight	Animal experiments were approved by the institutional ethics and animal welfare committee and the national authority according to §26ff. of Animal Experiments Act, Tierversuchsgesetz 2012 – TVS 2012 (license number BMWF 68.205/188-V/3b/2018, GZ-2021-0430018).

Note that full information on the approval of the study protocol must also be provided in the manuscript.

Flow Cytometry

Plots

Confirm that:

- [x] The axis labels state the marker and fluorochrome used (e.g. CD4-FITC).
- [x] The axis scales are clearly visible. Include numbers along axes only for bottom left plot of group (a 'group' is an analysis of identical markers).
- [x] All plots are contour plots with outliers or pseudocolor plots.
- [x] A numerical value for number of cells or percentage (with statistics) is provided.

Methodology

Sample preparation	Cell samples were washed and stained with Zombie Aqua™ viability dye (BioLegend, USA), followed by fixation in 2% ROTI®Histofix (Carl Roth, Germany) for 15 min. For cell permeabilization, samples were washed, re-suspended in 0.2%Triton X-100 (PanReac ApliChem, USA) in PBS + 10% FCS (FACS buffer) and incubated for 15 min. After incubation in 0.1% Triton-FACS buffer for 30 minutes, permeabilized cells were incubated with a primary antibody against ABCC1 (anti-MRP1, ab24102, lot: GR284248-19, Abcam, UK; dilution 1:200) followed by a secondary fluorescence-labelled antibody (Goat anti Mouse IgG, FITC, Cat-No: P31582, lot: 1915874, Thermo Fisher Scientific, Austria; dilution 1:200).<br><br>For competitive proliferation assays:<br>To assess the effect of CRISPR/Cas9-mediated gene targeting on cell proliferation, Cas9-expressing cells were transduced with lentiviral sgRNA/IRFP670 expression vectors and IRFP670-positive cells were monitored at regular intervals by flow cytometry.
Instrument	For intracellular staining: Stained samples were measured using a FACS Canto II flow cytometer (BD Biosciences), and analyzed with the FlowJo software (FlowJo, LLC).<br><br>For competitive proliferation assays: Cells were monitored at regular intervals using an IntelliCyt IQueScreener Plus (BioScience, Sartorius Group, Germany)
Software	FACSDIVA, FlowJo, ForeCyt
Cell population abundance	The proportion of living cells was always higher than 70%.
Gating strategy	For intracellular staining:<br>1.) FSC-area vs SSC-area was used to discriminate cells from debris.<br>2.) FSC-area vs width was used to discriminate singlets.<br>3.) ZombieAqua negative cells were used to discriminate live from dead cells<br>4.) ABCC1-PacificBlue+ cells were displayed to identify ABCC1 expression in WT vs ectopically ABCC1 overexpressing cells<br><br>For competitive proliferation assays:<br><br>1.) FSC-area vs SSC-area was used to discriminate cells from debris.<br>2.) FSC-area vs width was used to discriminate singlets.<br>3.) GFP+/APC+ cells were used to identify Cas9- and sgRNA-expressing cells.

- [x] Tick this box to confirm that a figure exemplifying the gating strategy is provided in the Supplementary Information.
